# Organizational effects of testosterone on the number of mating partners and reproductive success in females of a social rodent

**DOI:** 10.1038/s41598-025-03708-y

**Published:** 2025-07-01

**Authors:** Loreto A. Correa, Antonia Aspillaga-Cid, Juan D. Riquelme, Álvaro Ly-Prieto, Loren D. Hayes, Luis A. Ebensperger

**Affiliations:** 1 Escuela de Medicina Veterinaria, Facultad de Medicina y Ciencias de la Salud, Universidad Mayor, Camino la Pirámide, 5750, Huechuraba, Santiago Chile; 2 Departamento de Ecología, Facultad de Ciencias Biológicas, Pontificia Universidad Católica de Chile, Casilla 114-D, Santiago, Chile; 3 Cienciambiental Consultores S.A., Lo Encalada 266, Ñuñoa, Santiago Chile; 4 Departament of Biology, Geology and Environmental Sciences, University of Tennessee at Chattanooga, Chattanooga, TN 37403 USA

**Keywords:** Anogenital distance, Common degu, Litter size, Number of males, Number of mating partners, Serum testosterone

## Abstract

The timing of exposure to testosterone produces activational and organizational effects in vertebrates. Evidence in female birds and mammals suggests that high serum testosterone levels, and the exposure to high testosterone levels during early development, are negatively associated with female fertility and sexual attractiveness to males. We tested these hypotheses by examining associations between serum testosterone levels (an indirect index of activational effects), anogenital distance (AGD, a direct index of organizational effects), the number of male mating partners attained by females, and the number of offspring weaned. We examined 160 wild degu (*Octodon degus*) females during the mating season, and 308 females during the nursing season, across an 11-year study. During the mating season, we found no association between female serum testosterone levels or AGD on the number of male mating partners, and on the number of offspring weaned. However, we found that the number of male mating partners was positively associated with the number of offspring weaned, suggesting that females attain direct fitness benefits from polyandrous mating. During the nursing season, we recorded a positive association between female AGD and the number of offspring weaned. This indicates that testosterone organizational effects positively impact female fertility. This finding is unique and adds to the theoretical framework of female phenotypical masculinization via intrauterine position phenomenon.

**Supplementary Information:**

The online version contains supplementary material available at 10.1038/s41598-025-03708-y.

## Introduction

Although testosterone (T) is known as the “male hormone”, females also produce T, which is important for development and regulation of the female reproductive and central nervous systems, and for the normal functioning of muscle, bone, cartilage, adipose, and epidermal tissues^1–4^. Additionally, T regulates the aggressive and sexual behavior of female vertebrates^1,2,5^. Like other steroid hormones, T can trigger its biological effects through organizational and/or activational effects^6,7^. Organizational effects occur when circulating T binds to receptors during critical phases of development (perinatal and puberty), influencing the development of sexual characteristics that are irreversible and permanent. Activational effects occur during adulthood due to the activation of receptors by the binding circulating T, which influences sexual characteristics (mostly behavioral). Thus, activational effects are reversible and transient because they end when the hormone is metabolized or converted into an inactive form^6,7^. However, organizational effects may increase sensitivity to circulating T levels in adults, implying that activational effects are conditioned by organizational effects^6,7^. Since most T studies focus on males, the simultaneous examination of activational and organizational effects of T in females remains scarce. One exception is Goel and Bale^7^ study that experimentally examined the activational and organizational effects of T in the display of anxiety-like behaviors in female domestic mice (*Mus musculus*). Specifically, female mice administered with testosterone-propionate during postnatal development and puberty exhibited fewer anxiety-related behaviors across different tests compared to females that did not receive hormonal treatment. This reduction in anxiety-related behavior was interpreted as a behavioral masculinization of experimental females^7^.

High circulating T levels during the reproductive season (activational effects) have been generally associated with poor reproductive performance and decreased fertility in females^4,5,8^. Hormonal manipulations in dark-eyed juncos (*Junco hyemalis*) indicate that females with high T levels are less efficient in choosing a mating partner^2^, exhibit longer times to lay eggs^9^, and spend less time incubating clutches, all of which contribute to reduced reproductive success^10,11^. Similarly, zebra finch (*Taeniopygia guttata*) females with high circulating T levels produce relatively small clutches^12^, and bank vole (*Myodes glareolus)* females with high T levels exhibit fewer sexual partners than females with low T levels^13^. However, positive effects of elevated T have been reported in other species. For example, in some cooperatively breeding mammals (meerkats and mole rats), females with higher circulating T levels are socially dominant, facilitating the monopolization of within-group reproduction^14,15^. Thus, the effects of T on fitness tend to be complex and depend on the variables under investigation. Unsurprisingly, mixed results have been reported in some species. For example, in meerkats (*Suricata suricatta*), the beneficial effect of elevated T in social dominance and reproductive success^16,17^ is offset by the negative effect of mothers with elevated T on the offspring health and survivorship^17^. In rock hyraxes (*Procavia capensis*), females with high hair T levels exhibit lower copulation success and a lower probability to be mate-guarded by males compared with females with low hair T levels^18^. However, female hair T levels seem unrelated to the number of male mating partners^18^ and subsequent litter size^8^ in rock hyraxes.

In adult female mammals from litter-bearing species, the phenotypical gradient in genital masculinization represents direct organizational effects of T on female fetuses^19,20^ through the intrauterine position phenomenon (IUP)^19,20^. Specifically, female fetuses within a litter are exposed to androgens released from their male siblings in utero. A female fetus that develops between two male siblings is exposed to higher concentrations of T and thus develops masculinized traits. In contrast, a female fetus that develops without contiguous male fetuses or between two females is exposed to a relatively low concentration of T and thus develops into an adult female with exacerbated female traits. A female fetus that develops between one male and one female fetus experiences an intermediate concentration of T and develops into a female with typical female traits^19,20^. Thus, prenatal exposure to higher or lower concentrations of T may result in within-litter and population gradients of female offspring masculinization that irreversibly modify the phenotype and persist through adulthood^20,21^. A morphometric trait that varies with the exposure to T in utero is the length of anogenital tissue or anogenital distance (AGD). Because prenatal exposure to T affects the development of perineal tissue, the distance between the ventral commissure of the anus and the vaginal commissure is longer in females exposed to higher concentrations of T, and shorter in females exposed to lower T concentrations^19,20^. Thus, AGD is a direct index of fetal T organizational effects and allows a noninvasive assessment of female masculinization level^22^. Female AGD has been negatively associated with reproductive success, reproductive performance, and maternal behavior in several rodent species^19–21,23,24^, rabbits^25–27^, and domestic pigs^28^. However, neutral associations between female AGD and reproductive success have been similarly recorded in rodents^29–31^ and positive associations between female AGD and maternal behavior have been recorded in rodents and rabbits^21,27,31^. Thus, the relationship between AGD and female reproductive performance across mammals remains equivocal.

The common degu (*Octodon degus*) is a highly social rodent where individuals live in social groups that vary in size and individual sex composition^32,33^. Social group size ranges between 1–11 adults (mean = 3.7, SD = 2.0) during the mating season, which take place during the austral winter (May-Jul), and 1–8 adults (mean = 3.1, SD = 1.3) during the nursing season, which take place during austral spring (Sept-Nov)^34^. Females can be found in multifemale-multimale groups, unimale-multifemale groups, one female-one male pairs, and less frequently, as solitary individuals^35^. However, the most common social group composition is the multi-female social group, with and without males^36,37^. Group composition changes between seasons and years, in part due to high mortality rates of adults and social instability between seasons^38,39^. However, social group membership is stable within seasons^39^. Degu social groups are not kin structured, as the genetic relatedness within social groups is not different from that across the population^40^. Social group members cooperate through communally digging and sharing underground burrows^41,42^, and females in multi-female groups use these burrows to rear their offspring communally and exhibit allonursing^43^. During daytime, degus forage in groups characterized by fission–fusion dynamics, meaning that individuals leave and join different groups within seconds or minutes^44–46^. Degus mate during June (austral winter) and after a gestation period of 87 ± 3 days^47^, females give birth to an average of 3.42 ± 2.71 (SD) precocial offspring, with a range of 1–10^33^. Lactation is relatively short, lasting approximately 30 days (4–5 weeks)^48^ during Sept-Oct (austral spring). Interestingly, male and female degus mate with multiple partners from the same or different social groups, and the number of mating partners is positively associated with reproductive success in males^33,49^, but not in females^33^. During the mating season, females move as much as males, which makes their captures less frequent (Aspillaga-Cid, unpublished data). On the contrary, during the nursing season, lactating females remain in their burrows and go out to feed daily, which makes their capture more frequent. Additionally, free-living females exhibit a gradient in AGD^22,31^ linked to the intrauterine position where each female developed^22^. Thus, variability in female degu AGD represents a direct index of fetal T organizational effects^22^. Previous studies in degus indicate a positive association between female body weight and the number of offspring weaned, and between female AGD and offspring’s body weight at weaning^31,35^. Most intriguingly, AGD female composition within free-living groups affect positively the number of offspring weaned. Individual females wean more offspring when in social groups where most females exhibit relatively long AGD^35^, probably because during nursing, long AGD mothers make a higher maternal investment^50^. The relationship between female AGD and the number of male mating partners has not been studied in degus or other species. In female degus, serum T levels have been analyzed in three previous studies^22,51,52^, but only one examined the potential effect of T on maternal behavior. Specifically, Ebensperger et al.^51^ reported that maternal T levels are negatively associated with the offspring grooming frequency, where high T mothers groom their offspring at lower frequencies than low T mothers. None of these studies examined the association between serum T and the number of male mating partners, nor the association between this hormone and female reproductive success. In adult female degus, T organizational and activational effects are decoupled, as AGD and serum levels are not associated^22,42^.

In this study, we analyzed two independent data sets. The first data set included 160 mated females with data recorded during the mating season exclusively, and a second included 308 mated females with data recorded during the nursing season exclusively. We considered these two data sets because most of our animals were captured only in one season of the year (mating or nursing) due to high adult mortality^33^. Using a unique database only by mated females captured during both seasons of the year would have reduced our sample size to 125 females. Our first objective was to examine the potential association between female AGD, adult female serum T levels, and the number of males within social groups (a measure of mating opportunities), and their factor interaction, on the number of male mating partners attained within, outside the social group, and in total, during the mating season. We hypothesized that the effects of female AGD and adult serum T levels on the number of male mating partners attained within and outside the social group are contingent on the number of males within the social group (hypothesis 1, Table 1). We predicted that during the mating season, females with shorter AGDs, lower serum T levels, and that were members of social groups with several males would (i) attain more mating partners in total and (ii) more mating partners within the social group than females with longer AGDs and higher serum T levels, who were members of social groups with few or no male group mates. We also predicted that during the mating season, females with shorter AGDs, lower serum T levels, and who were nesting solitarily or were members of social groups with few males would (iii) have more mating partners outside the social group than females with longer AGDs, higher serum T levels, and that were members of groups with many males. We used the mating season data set to examine predictions i, ii, and iii. Our second objective was to determine the potential influence of female AGD, adult female serum T levels, and the number of male mating partners attained during mating season on female litter size at weaning. Litter size can only be determined at weaning during the nursing season, as mothers give birth inside the burrow where we could not access. After weaning, the offspring begins to leave burrows. Based on previous observations with degus in captivity, supporting that female AGD and litter traits are not associated^50^, we hypothesized that female AGD, female serum T levels, and the number of male mating partners attained by mated females during the mating season are not associated with litter size at weaning (hypothesis 2, Table 1). Thus, we predicted that mated females exhibiting short or long AGDs, low or high serum T, and that mate with few or several males during the mating season would (iv) wean litters of similar size. To examine prediction (iv) we used the mating season data set, except for the response variable (litter size at weaning) obtained at the end of the nursing season. Our third objective was to examine the potential association between female AGD and serum T during the nursing season and litter size at weaning. In this objective, we were only interested in knowing how female individual traits during the nursing could affect litter size at weaning. Based on previous observations, where long AGD females wean heavier and more offspring if they nurse together in the same social group, and where high T mothers are less attentive to the offspring, we hypothesized that female AGD and serum T levels interact to explain litter size at weaning (hypothesis 3, Table 1). We predicted that (v) females with long AGDs and low T levels would wean more offspring than females with short AGDs and high T levels. To examine prediction (v) we use the nursing season data set.

**Table 1:** Full statistical models examined.

Model	Hypothesis and predictions	Data set	Response variable	Fixed factors	Interaction	Random factors	No of replicates
1	Hypothesis 1, prediction i	Mating season	Total no of male mating partners	Focal female AGD + focal female serum T + focal female body weight + no of males within social group	(Focal female AGD x focal female serum T x n° of males within social group)	Year + SGID + female ID	n = 160. Include all females studied during mating season, that in spring weaned at least one offspring
2	Hypothesis 1, prediction ii	Mating season	No of male mating partners within social group	Focal female AGD + focal female serum T + focal female body weight + no of males within social group	(Focal female AGD x focal female serum T x n° of males within social group)	Year + SGID + female ID	n = 118. Include all females studied during mating season, that were members of social group with at least one male member, and that in spring weaned at least one offspring
3	Hypothesis 1, prediction iii	Mating season	No of male mating partners outside social group	Focal female AGD + focal female serum T + focal female body weight + no of males within social group	(Focal female AGD x focal female serum T x n° of males within social group)	Year + SGID + female ID	n = 160. Include all females studied during mating season that in spring weaned at least one offspring
4	Hypothesis 2, prediction iv	Mating season	Litter size at weaning	Focal female AGD + focal female serum T + focal female body weight + no of male mating partners attained	(Focal female AGD x focal female serum T x n° of male mating partners attained)	Year + SGID + female ID	n = 160. Include all females studied during mating season that in spring weaned at least one offspring
5	Hypothesis 3, prediction v	Nursing season	Litter size at weaning	Focal female AGD + focal female serum T + focal female body weight	(Focal female AGD x focal female serum T)	Year + SGID + female ID	n = 308. Include all females studied during nursing season

AGD = anogenital distance, T = testosterone, SGID = social group identity, female ID = female identity. Study year, SGID and female.

ID, were random factors. All other factors were fixed. Models 1, 2, 3 and 4 were run with the mating season data set. Model 5 was run with the nursing season data set.

## Results

### Mating season

#### Descriptive results

During the mating season, we studied 160 mated females that weaned at least one offspring in spring. Of these, ten females were living solitarily, 32 were in social groups without males, and 118 were in social groups with at least one male group member (1–5). The total number of male mating partners (including within and outside the social group) attained by the females ranged from one to six (mean: 2.08 ± 1.34). Of the 118 females that had at least one male group member, 56 mated only with males (1–5) from a different social group, 20 mated only with males (1–2) from the same social group, and 42 mated both with males from the same (1–2) and with males from other social groups (1–5). The 42 females that were either solitary or without male group members mated with 1 to 5 males from other social groups. Genetic analyses of maternity and paternity revealed that females studied during the mating season (n = 160) weaned 4.68 ± 2.03 offspring (range: 1–10).

#### Total number of male mating partners

**Fig. 1:**
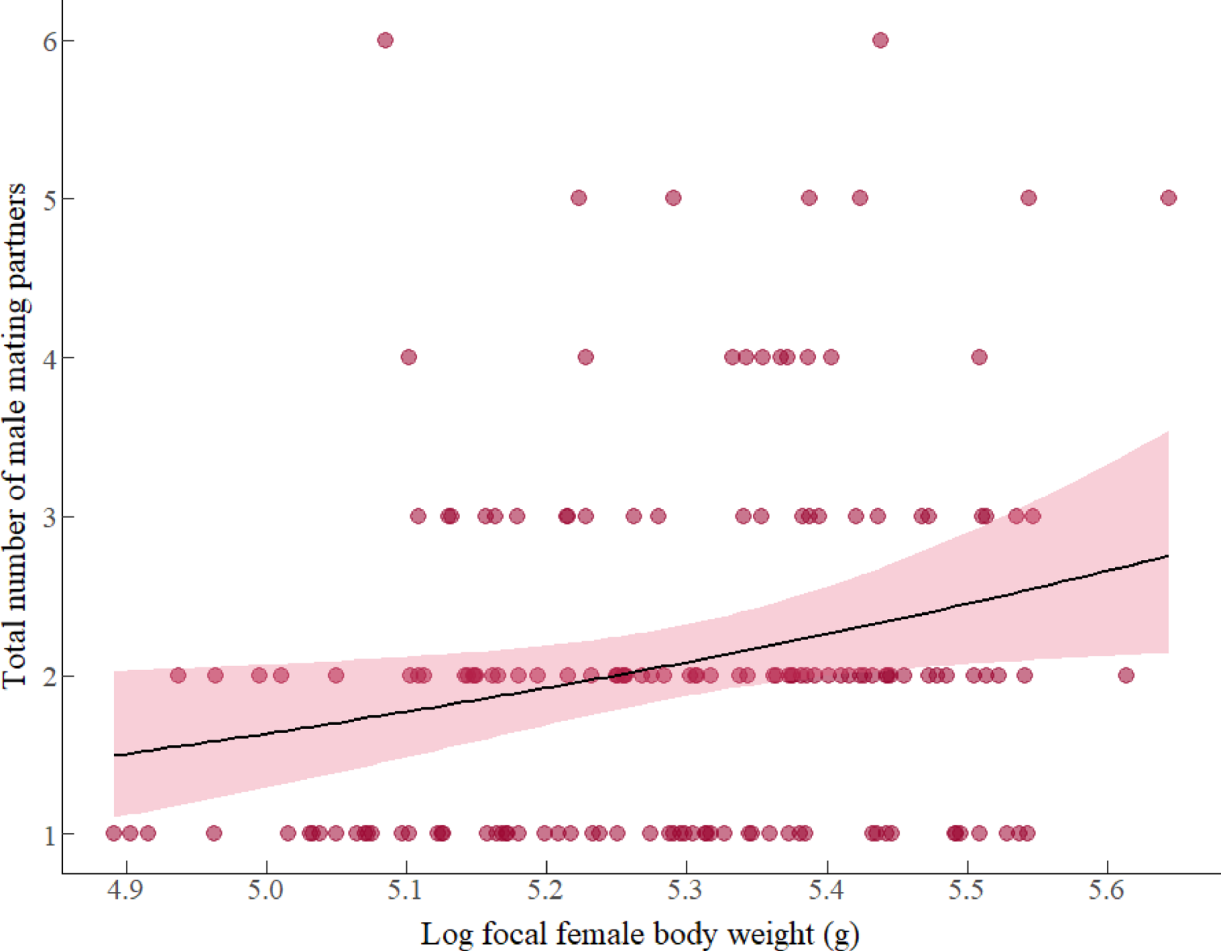
Association between the log10-transformed of focal female body weight (g) and the total number of male mating partners attained during mating season. The positive association indicates that heavier females mated with more males than lighter females. Circles represent the total of 160 females that served as replicates and the pink region indicates 95% confidence intervals fitted with a Poisson error distribution with a log-link function.

Results from model 1 indicated that female body weight during the mating season had a positive effect in the total number of male mating partners attained (Table 2, model 1). This suggests that heavier females mated with a larger number of males than lighter females (Fig. 1), where for every unit increase in log-transformed body weight, the total number of male mating partners attained increased by a factor of 2.24 males (β: 0.806, *e*^0.806^ = 2.24). Neither female AGD, female serum T levels, the number of males within the social group, nor the interaction between these factors predicted the total number of male mating partners attained during the mating season (details in table S1, supplementary material 1, model selection).

**Table 2:** Results of models 1, 2, 3, 4 and 5 selected after model selection routine from full models.

Random effects	Variance	Standard deviation		
Model 1: total number of male mating partners attained (mating season dataset) n = 160 mated female replicates studied during winter				
Year	0.001	0.032		
Social group ID	0.000	0.000		
Female ID	0.000	0.000		

Fixed effects	Estimate*	Standard error	z value	*p* value
Intercept	0.723	0.059	12.20	< 0.001
Log focal female body weight	0.806	0.353	2.280	0.023

Random effects	Variance	Standard Deviation		
Model 2: number of male mating partners attained within social group (mating season dataset) n = 118 mated female replicates studied during winter				
Year	0.026	0.160		
Social group ID	0.013	0.113		
Female ID	0.000	0.000		

Fixed effects	Estimate*	Standard error	t value	*p* value
Intercept	− 0.625	0.169	− 3.703	< 0.001
Log focal female body weight	1.373	0.862	1.593	0.111
no of males within social group	0.270	0.112	2.421	0.016

Random effects	Variance	Standard deviation		
Model 3: number of male mating partners attained outside social group (mating season dataset) n = 160 mated female replicates studied during winter				
Year	0.0212	0.143		
Social group ID	0.000	0.000		
Female ID	0.000	0.000		

Fixed effects	Estimate*	Standard error	t value	*p* value
Intercept	0.448	0.085	5.258	< 0.001
Log focal female body weight	0.670	0.425	1.578	0.115
No of males within social group	− 0.163	0.068	− 2.406	0.016

Random effects	Variance	Standard deviation		
Model 4: litter size at weaning (mating season dataset) n = 160 mated female replicates studied during winter				
Year	0.000	0.000		
Social group ID	0.001	0.024		
Female ID	0.000	0.000		

Fixed effects	Estimate*	Standard error	z value	*p* value
Intercept	1.526	0.037	40.471	< 0.01
Log focal female body weight	0.642	0.240	2.672	0.008
Total no of male mating partners	0.113	0.031	3.647	< 0.01

Random effects	Variance	Standard deviation		
Model 5: litter size at weaning (nursing season dataset) n = 308 mated female replicates studied during spring				
Year	0.025	0.157		
Social group ID	0.000	0.000		
Female ID	0.059	0.243		

Fixed effects	Estimate*	Standard error	z value	*p* value
Intercept	1.317	0.061	21.505	< 0.01
Log focal female body weight	1.565	0.292	5.351	< 0.01
Focal female AGD	0.155	0.075	2.058	0.040

The table contains the results for each of the five models. Each model includes the predictors retained from the best model after model selection routines from full models described in Table 1. All models were fitted with generalized mixed effect models with Poisson distribution and log (link) function, and continuous predictors were mean-centered prior analysis.

Model 1: *Expected total number of male mating partners attained for a female with an average Log body weight (β_0_) = *e*^0.723^ = 2.06; Model 2: *Expected number of male mating partners attained within social group for a female with an average Log body weight, and males within the social group (β_0_) = *e*^-0.625^ = 0.54; Model 3: *Expected number of male mating partners attained outside social group for a female with an average Log body weight, and males within the social group (β_0_) = *e*^0.448^ = 1.57; Model 4: *Expected litter size at weaning for a female with an average Log body weight, and number of male mating partners attained (β_0_) = *e*^1.526^ = 4.60; Model 5: *Expected litter size at weaning for a female with an average Log body weight and AGD (β_0_) = *e*^1.317^ = 3.73.

#### Number of male mating partners within the social group

**Fig. 2:**
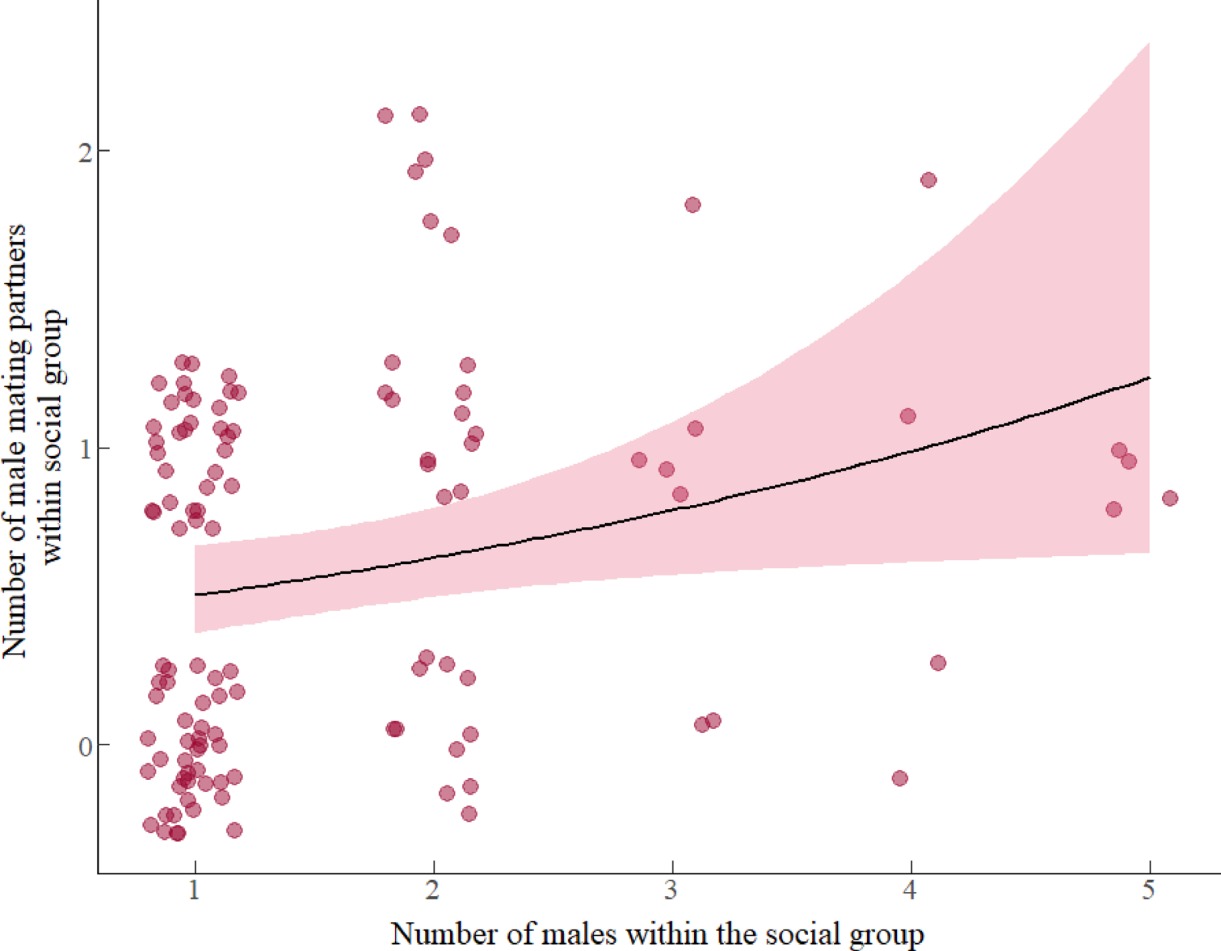
Association between the number of adult males within the social group and the number of male mating partners attained within-group by the females during the mating season. Circles represent the total of 118 focal females that served as replicates and the pink region indicates 95% confidence intervals fitted with a Poisson error distribution with a log-link function.

Results from model 2 revealed a positive association between the number of males within the social group and the number of male mating partners attained within the social group (Table 2, model 2). Mated females that were members of social groups with more males had more male mating partners within the social group (Fig. 2). Specifically, the addition of one male within the social group increased the number of male mating partners attained within social group by a factor of 1.31 males (β: 0.270, *e*^0.27^ = 1.31). Neither female AGD, female serum T level, female body weight, nor the interaction between factors predicted the number of male mating partners within the social group during the mating season (details in table S2, supplementary material 1, model selection).

#### Number of male mating partners outside the social group

**Fig. 3:**
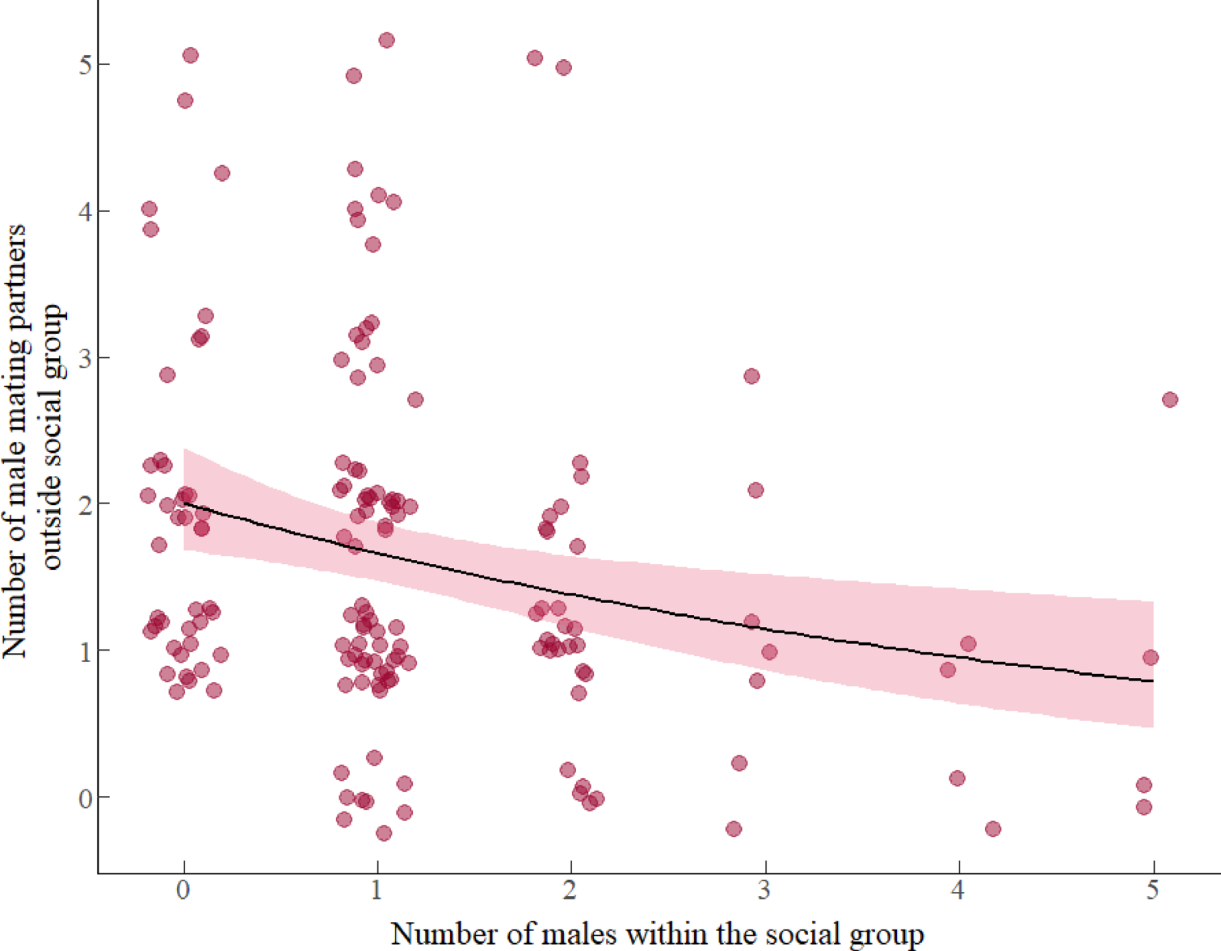
Association between the number of adult males within the social group and the number of male mating partners attained by females from outside the social group during mating season. Circles represent the total of 160 focal females that served as replicates and the pink region indicates 95% confidence intervals fitted with a Poisson error distribution with a log-link function.

Results from model 3 revealed a negative association between the number of males within the social group and the number of male mating partners attained by the females outside the social group (Table 2, model 3). Mated females that were members of social groups with fewer males mated with a larger number of males outside the social group (Fig. 3). The addition of one male within the social group decreased the number of male mating partners attained within social group by a factor of 0.85 males (β: -0.163, *e*^-0.163^ = 0.85). Neither female AGD, female serum T levels, female body weight, nor the interaction between factors predicted the number of male mating partners outside the social group during the mating season (details in table S3, supplementary material 1, model selection).

#### Litter size at weaning

**Fig. 4:**
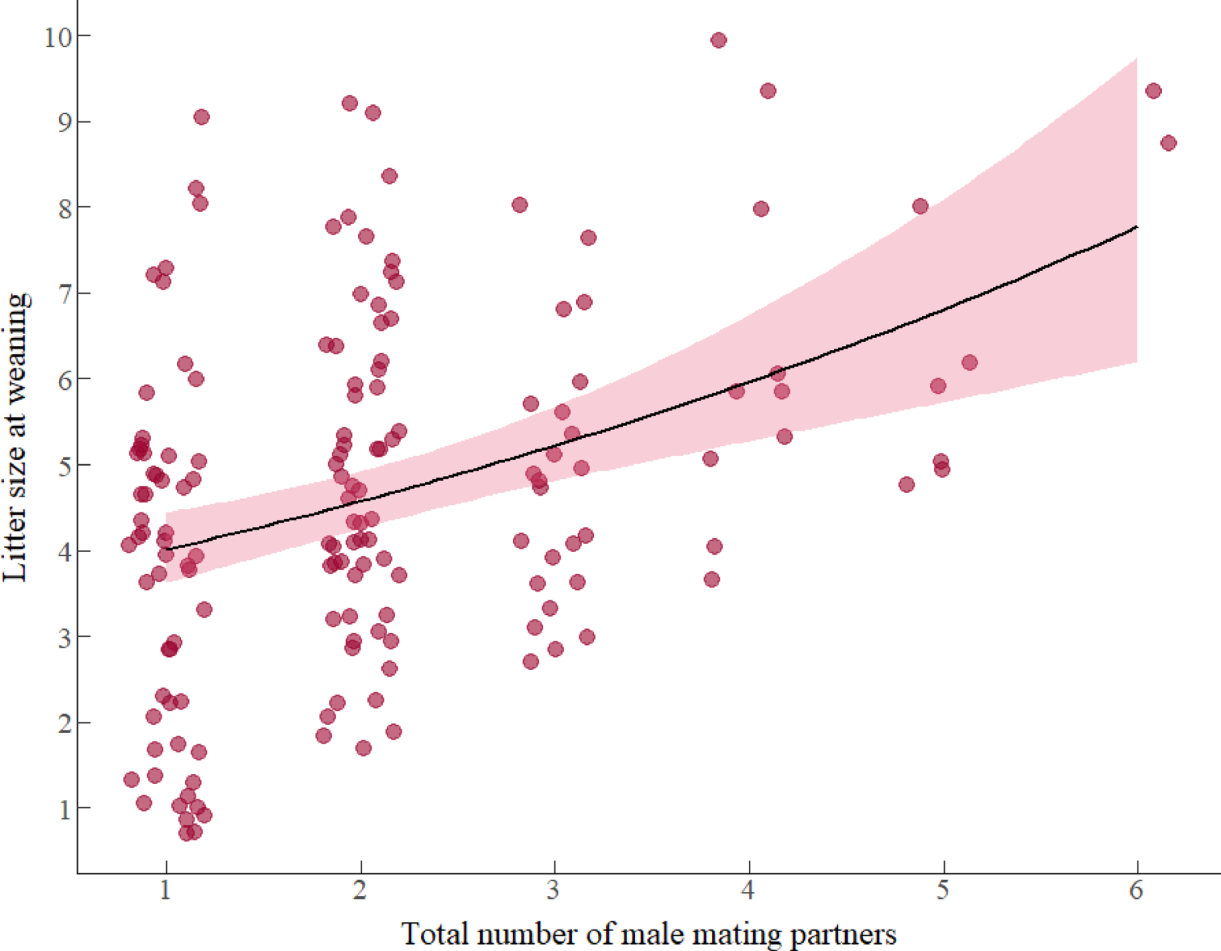
Association between litter size at weaning and total number of male mating partners attained by females during the mating season. Circles represent the total of 160 females that served as replicates and the pink region indicates 95% confidence intervals fitted with a Poisson error distribution with a log-link function.

Results from model 4 revealed positive associations between female body weight and the number of mating partners attained during the mating season, with the number of offspring weaned (Table 2, model 4). Mated females that were heavier during the mating season weaned more offspring during the nursing season, where for every unit increase in log-transformed body weight, the litter size at weaning increased by a factor of 1.90 offspring (β: 0.642, *e*^0.642^ = 1.90). Additionally, females that mated with more males weaned more offspring (Fig. 4). Specifically, the increase in one unit of the number of male mating partners attained, the litter size at weaning increased by a factor of 1.12 (β: 0.113, *e*^0.113^ = 1.90). Neither female AGD, female serum T levels, nor the interaction between factors predicted the number of offspring weaned (details in table S4, supplementary material 1, model selection).

### Nursing season

#### Descriptive results

We studied 308 mated females during the nursing season. Of these, 243 females were members of multi-female or multi-male/multi-female social groups, 43 were in unifemale-multimale social groups, and 22 females were solitary living individuals. Genetic estimates of maternity indicated that 275 females weaned at least one offspring, and that 33 females weaned zero offspring. The litter size at weaning averaged 4.57 ± 2.12 and ranged from 1 to 10 offspring (n = 275 reproductively successful females).

#### Litter size at weaning

**Fig. 5:**
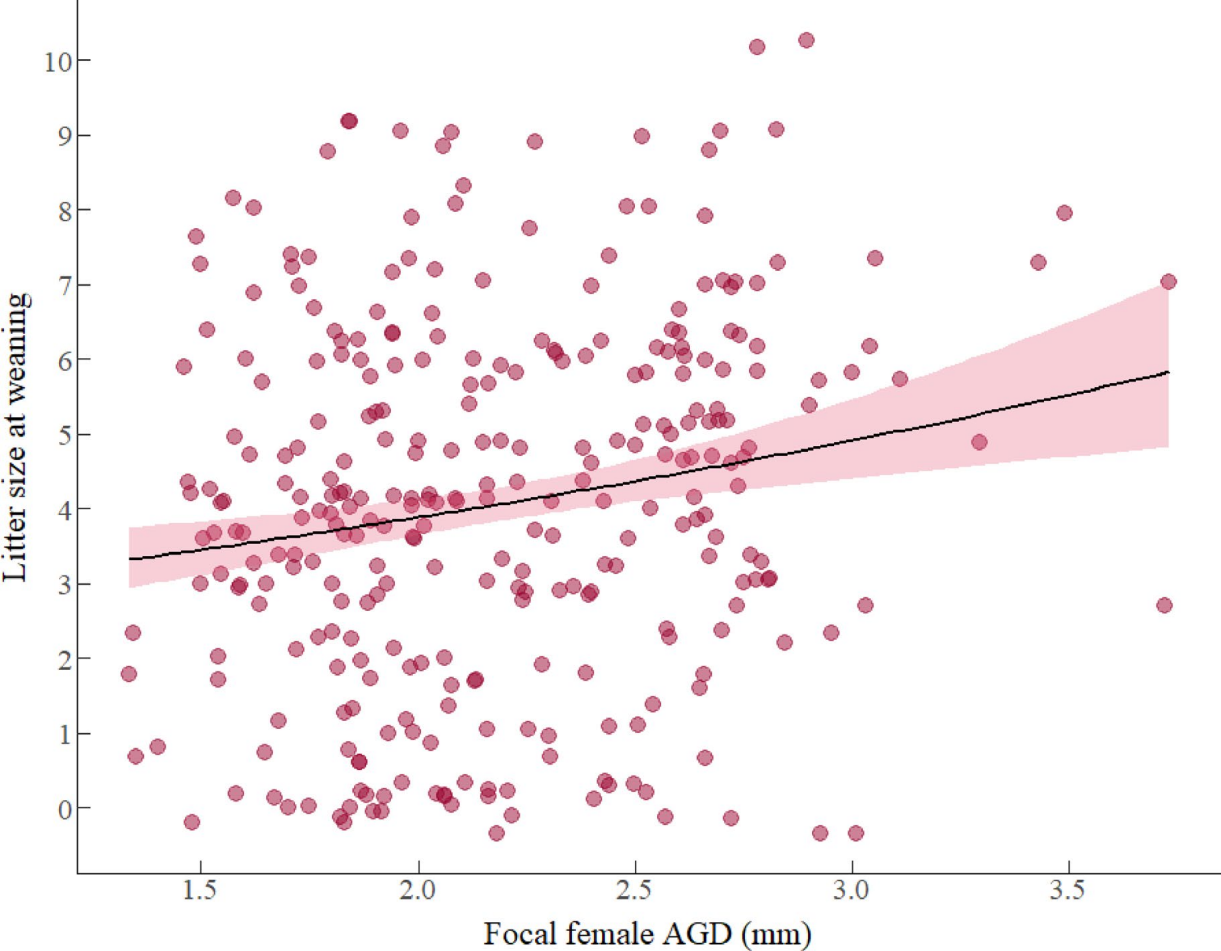
Association between litter size at weaning of focal females and focal female AGD (mm) during the nursing season. Circles represent the total of 308 females that served as replicates and the pink region indicates 95% confidence intervals fitted with a Poisson error distribution with a log-link function.

Results from model 5 revealed a statistically significant and positive association between female body weight during the nursing season and the number of offspring weaned, implying that heavier females weaned more offspring (Table 2, model 5). For every unit increase in log-transformed body weight, the litter size at weaning increased by a factor of 4.78 offspring (β: 1.565, *e*^1.565^ = 4.78). Additionally, we found a statistically significant and positive association between focal female AGD during the nursing season and the number of offspring weaned; thus, long AGD females weaned more offspring than short AGD females (Table 2, model 5, Fig. 5). For every unit increase AGD, the litter size at weaning increased by a factor of 1.17 offspring (β: 0.155, *e*^0.155^ = 1.17). Neither female serum T level, nor the interaction between factors predicted the number of offspring weaned (details in table S5, supplementary material 1, model selection).

## Discussion

Our results revealed that female serum T levels (an indirect measure of activational effects of T) and female AGD (a direct index of organizational effects of T) were not associated neither with the total nor with within or outside group male mating partners attained. Thus, we did not support that females with long AGDs or with higher T levels are less successful at finding and attracting mating partners. The absence of these effects agrees with results from female rock hyraxes, where T levels are not associated with the number of mating partners^18^, but oppose to findings in female bank voles, where females exhibiting high T levels attain fewer mating partners than low T females^13^. Our results also departed from findings in domestic mice and European rabbits, where long AGD females are less sexually attractive to males who prefer to mate with short AGD females^19,25^. Female body weight during mating was the only factor associated with the total number of male mating partners attained by female degus. A similar finding was reported in the Mediterranean flour moth (*Ephestia kuehniella*), in which heavier females have a higher probability to re-mate with a different male than lighter females^53^. Additionally, our findings are in agreement with findings in modern humans (*Homo sapiens*), where underweight women report fewer sexual partners than near average weight and overweight women^54^. Comparative studies across mammals have further examined the correlation between female body weight during the reproductive season and litter size^55,56^. Findings indicate that this association is positive in some species, but also negative or unexistent^55,56^. In degus, we have shown that heavier females wean larger litters during nursing season^31,35^, a finding that mirrors our current result revealing that female body weight during the mating season is positively associated with litter size. Because males could increase their reproductive success by mating with more fertile females, we cannot rule out that male degus prefer heavier females. This could explain why heavier females are more polyandrous than lighter females. In birds and mammals, body weight and age are correlated, and an association between age and polyandry levels has been described^57^. In degus, we did not examine the potential association between female age and polyandry level, because, in our population, 85% of adult females do not survive to the second year of life^33^.

Polyandry is a common mating system in female vertebrates and invertebrates^58–61^. Avise and Liu^58^ examined polyandrous mating in 49 mammal species, and reported that 40% of litters involve multi-paternity and that were sired by a mean of 1.5 males^58^. Thus, female degus are relatively more polyandrous than other female mammals, with 64.3% of litters exhibiting multi-paternity and involving a mean of 2.1 males. Results from model 4 indicate that the number of male mating partners attained by females during the mating season is positively associated with litter size at weaning, suggesting that female degus could experience direct fitness benefits from polyandrous mating. A recent performed a meta-analytic approach^61^ based on 77 animal species (from worms to mammals) confirmed that females attain direct fitness benefits from multiple mating^61^. For example, in females of Trinidadian guppies (*Poecilia reticulata*), the clutch size is larger in polyandrous relative to monandrous females^62^, while in the yellow-toothed cavy (*Galea musteloides*) and the brown antechinus marsupial (*Antechinus stuartii*), litter size at weaning is larger in females that breed with several males than in females that breed with one male^63,64^. Experimental studies in invertebrates similarly support direct fitness benefits of polyandrous mating in the females. For example, in ladybirds (*Coccinella septempunctat*a), pseudoscorpions (*Cordylochernes scorpioide*), field crickets (*Gryllus bimaculatus*), and the Wellington tree wētā (*Hemideina crassidens*) females that mate with two or more males lay more eggs^65^, produce more offspring during their lifetime^66^, and attain higher hatchling success^67,68^ relative to females that mate with a single male. Aside from the potential fitness benefits that females may attain by mating with several males, the positive association between the number of male mating partners and litter size is expected because the probability of multi-paternity is greater in females that produce larger litters^58,59^. Therefore, this result must be interpreted with care. Overall, we now have an adequate understanding of the consequences of female polyandrous mating in degus, but are far from understanding the underlying proximal mechanisms involved. Future laboratory and seminatural studies are needed to examine how (i) good genes, (ii) genetic by environment interactions, and (iii) genetic incompatibility avoiding mechanisms^69^ provide suitable explanations for this mating behavior.

Our current findings further confirmed our previous findings based on a smaller number of study years, that females and males mate with sexual partners from the same and different social groups^33^, and that the number of sexual partners attained outside the social groups increases when the mate availability within the social group is low or zero^49^. All together, these findings confirm that in degus the social group is a main source of potential mates, but that social groups do not represent reproductive units. Similar results have been recorded in the striped mouse (*Rhabdomys pumilio*) where females mate with the territorial male of the same social group (64% of offspring paternity), but also with males of neighboring social groups (28% of offspring paternity)^70^. However, in degus, polyandrous mating behavior is more frequent than in striped mice, maybe because male degus are not territorial^35,49^, and because during the mating season the females move as much as the males (Aspillaga-Cid, unpublished data).

Female phenotypical masculinization has been associated with lower fertility based on the observations that masculinized females of Mongolian gerbils (*Meriones unguiculatus*) and domestic rabbits (*Oryctolagus cunniculus*) deliver smaller litters than feminized females^21,25,26^. Additionally, long AGD female (i.e., masculinized or exposed to more T in utero) rabbits, domestic pigs, and rodents experience numerous reproductive disadvantages, such exhibiting later puberty, longer and irregular estrous cycles, decreased likelihood of being impregnated, late breeding during the reproductive season, shorter reproductive lifespans, produce and detect fewer sexual cues, have less likely to wean offspring, and are less willing to nurse own offspring^19,21,23–28,30,71^. These findings contrast with the few other studies supporting reproductive advantages in long AGD females (masculinized or exposed to more T in utero). For example, long AGD females of Mongolian gerbils, domestic mice, rabbits, and degus display higher quality or greater quantities of maternal care^19,21,27,31^, and masculinized females of alpine marmots (*Marmota marmota*) are socially dominant over feminized females, a condition needed for breeding in this species^72^. Thus, degus seem unique in that long AGD females are reproductively more successful than short AGD females (i.e., feminized or exposed to less T in utero). Data from a wild population studied continuously for 11 years indicate that during nursing, but not during mating season, AGD is an important individual factor, since long AGD female degus wean more (this study) and heavier offspring^31,35^. We hypothesize that the positive association between AGD during the nursing season and litter size at weaning is the consequence of high maternal investment provided by long AGD mothers to the offspring^31,35,50^, and not the consequence of differences in fertility between females of different AGDs. During this study we recorded that the magnitude of the effect of focal female AGD on the number of offspring weaned is relatively large, since a female with a 3.55 mm AGD tends to wean 2.34 more offspring than a female with a 1.55 mm AGD. Altogether, our findings do not support maladaptive effects of T exposure during prenatal development in female degus. Instead, exposure to high T concentrations during prenatal development is associated with direct fitness benefits in females.

Evidence from our long-term study on a wild population of degus suggests that neither high levels of circulating T in adulthood nor prenatal exposure to high levels of T negatively affect fertility or the ability of females to find and attract mates. Most importantly, during the nursing season we found a positive association between female AGD and the number of offspring weaned by the females. To our knowledge, this study is the first to report fitness advantages for long AGD females. We additionally found that heavier females attain more sexual partners and wean more offspring than lighter females. Thus, female body weight during the mating and nursing seasons remains the most important aspect of phenotype influencing direct fitness, a finding consistent with previous evidence in vertebrates and invertebrates. Additionally, we found that polyandry is more frequent than monoandry in female degus, and that polyandrous mating contributes to enhance direct benefits in females. Evidence from invertebrates and vertebrates suggests polyandrous mating is a common behavior in the females, which is associated with indirect and direct fitness benefits, confirmed by our results.

## Methods

### Study population

Data came from a long-term study conducted between 2009 and 2019 (11 generations) on a natural degu population located at Estación Experimental Germán Greve Silva (33°23′ S, 70°31′ W, altitude 495 m), a field station of the Universidad de Chile. This study area is characterized by a Mediterranean climate with cold, wet winters and warm, dry summers. The sampling site consisted of open areas with scattered scrubs (*Proustia pungens*, *Vachellia caven*, and *Baccharis spp*.) and several herbaceous species (e.g., *Erodium spp*., *Senecio adenotrichius*). The total area examined was 2–3 ha and did not vary between years of study.

### Live trapping and telemetry

Annually, we conducted live trapping and radio-tracking to determine degu identity, phenotype based on AGD, and social group membership during the austral winter (mating season) and the austral spring (nursing season). Live trapping and telemetry during the mating season were conducted from the first week of May through the last week of July. Mating activity is synchronous and peaks during the last two weeks of June. We carried out a long period of trapping and telemetry to habituate degus to traps, because the peak of mating activity can be advanced or delayed depending on the amount of autumn rainfall^37^. Live trapping and telemetry during the nursing season were conducted from the last week of August through the first week of November. We chose this lengthy monitoring period because even though births typically are synchronous and concentrated during September, this life history schedule may change with year-specific abundance of food^37^. Additionally, we needed to trap the offspring, which became trappable at weaning (30 days after the birth). Trapping during nursing season ended when the daily capture rate of new offspring was less than 5% of all offspring captured^35^.

Degus are diurnally active and remain in underground burrows overnight^32^. A burrow system was defined as a group of burrow openings surrounding a central location spanning 1–3 m in diameter where individuals were repeatedly found during night-time telemetry^73^. Ten traps (Tomahawk model 201, Tomahawk Live Trap Company, Tomahawk, WI) were used at each burrow system daily. Traps were set prior to the emergence of adults during morning hours (06:00 h). Traps were baited with rolled oats, placed directly on the treadle that the animals step on to activate trap door closure. After 1.5 h, traps were closed until the next trapping day. The identity, location, sex (degus were sexed morphologically for AGD length, genital papilla size, and presence of a vaginal commissure), body weight (weighed to the nearest 0.1 g), and AGD were determined for all captured degus. At first capture, each degu received ID-coded tags on each ear (Model 1005–1, National Band and Tag Co., Newport). Adults weighing more than 130 g were fitted with 6–7 g radio-collars (AVM Instrument Co., Colfax, CA) with unique pulse frequencies. Previous studies at Estación Experimental Germán Greve Silva confirmed that night-time locations represent underground nest sites^32^. Locations were determined once per night approximately 1 h before sunrise using LA 12-Q receivers (for radio collars tuned to 150.000–151.999 MHz frequency; AVM Instrument Co., Auburn, CA) and handheld, 3-element Yagi antennas (AVM Instrument Co., Auburn, CA). The number of burrow systems monitored for each year, the number of days that each burrow system was trapped per year, and the number of radio-collared degus per year are reported in table S6, supplementary material 2.

### Social group determination

In this study, we used two complementary methods to quantify the size of social groups and to determine which individuals were members of the same social group. Degus group naturally and individuals of the same social group share the same burrow at night. To determine which individuals shared the burrow at night, we employed 1) burrow trapping during early morning activity and 2) radiotelemetry tracking during the night-time. To determine group composition, we first compiled a symmetric similarity matrix of pairwise associations of burrow locations of all adult degus during trapping and telemetry^74^. The association (overlap) between any two individuals was determined by dividing the number of early mornings that these individuals were captured at or tracked with radiotelemetry to the same burrow system, by the number of early mornings that both individuals were trapped or tracked with radiotelemetry on the same day^32,38^. To determine social group composition, a hierarchical cluster analysis of the association matrix was conducted using SOCPROG software^75^. The fit of the data was analyzed using a cophenetic correlation coefficient, and correlations between the actual association indices and the levels of clustering in the diagram. In this procedure, values above 0.8 indicate that the hierarchical cluster analysis provided an effective representation of the data^74^. The maximum modularity criterion^76^ was used to cut off the dendrogram and define social groups (further details about methodology to determine social group, and a dendrogram image (Fig. S1) are provided in supplementary material 3). We conducted separate determinations of social groups during the mating season and during the nursing season of each study year. We used individual group composition during the mating season to quantify the number of males within the social group, a measure of mate availability in social groups.

### Sample size

We examined two independent datasets: one based on the mating season (a life-history stage overlapping early winter) and another based on the nursing season (a life-history stage overlapping early spring). The mating season data set was used to examine predictions i, ii, iii and iv, and the nursing season dataset to examine prediction v. Our dataset during the mating season included n = 160 mated females that weaned at least one offspring. Of these 160 female replicates, 137 were captured only during one mating season at 9 months of age during the 10 years of our study, implying they were sampled once (no data were collected during the mating season in 2009). Ten females were captured during two consecutive mating seasons at 9 and 18 months of age and were sampled twice. One female was captured during three consecutive mating seasons at 9, 18 and 27 months of age, and therefore was sampled three times. For these 11 females captured and sampled during two or three consecutive mating seasons, each capture and T measure were considered statistically independent events because the social group and environmental conditions differed among years^22,31,35^.

The dataset during the nursing season included n = 308 mated females. Of these, 231 females were captured during one nursing season at 12 months of age, and thus were sampled once. A total of 34 females were captured during two consecutive nursing seasons at 12 and 24 months of age, and therefore were sampled twice. Three females were captured in three consecutive nursing seasons at 12, 24 and 36 months of age, and therefore were sampled three times. For these 37 females captured and sampled during two and three consecutive mating seasons, each capture and T measure were similarly treated as statistically independent events because the social group and environmental conditions differed between years^22,31,35^. All females had estimates of AGD and T measures.

### Individual variables: female AGD and female body weight

We quantified AGD (mm) to index individual phenotype in terms of masculinization of all adult and mated females with a digital caliper (precision 0.1 mm) at every capture event (Fig. S2, supplementary material 4). We only measured AGD in females exhibiting non-perforated vaginas (perforated vagina is an indicator of either estrus or recent parturition; degu females exhibit closed vaginas during all other life-history stages). All AGD measurements were taken by the same observer (LAC) across all eleven years. We calculated mean degu AGD from 18.19 ± 10.26 measurements per female (range: 2–61, n = 4,650 measurements from 468 female replicates), resulting in a single AGD estimate per female^22,31,35^. Intra-season repeatability of female AGD was 0.88 (measurement error = 0.12, n = 1,417 measurements) among the 185 females examined during only one season (winter or spring). Inter-season repeatability of female AGD was 0.93 (measurement error = 0.07, n = 2,160 measurements) among the 125 females examined in both seasons (winter and spring) within a same study year. Inter-year repeatability of female AGD was 0.94 (measurement error 0.06, n = 1,073 measurements) in the 37 females sampled in three consecutive seasons (winter and spring). These data suggest that female AGD is a stable measurement within and between individuals. In wild degus, as in domestic mice, the intrauterine position phenomenon (IUP) between one male and one female is the most frequent; 60–65% of the fetuses were in this IUP. The other two IUPs (between two males, or without contiguous males or between two females), are less frequent and are represented in the same proportion (~ 15–20% of individuals per IUP type^22,77^). This representation of each AGD phenotype matches a normal distribution with limits of ± 1 SD. In wild female degus, AGD distribution has a mean = 2.10 mm (SD = 0.54). Thus, we identified short AGD females as those one standard deviation below the mean (≤ 1.55 mm AGD), long AGD females as those one standard deviation above the mean (≥ 2.65 mm), and intermediate AGD females as those within one standard deviation of the mean (between 1.56–2.64 mm)^22,31,35^ (Fig. S3, supplementary material 4). This classification is only to describe different AGD phenotypes when discussing our results, as AGD was used as a continuous predictor for all statistical analyses. The mean AGD of each female was included in our analyses as the *focal female AGD*.

Females were weighed each time they were captured. The mean body weight (in grams) of each female in each season was determined by averaging weights recorded during mating and nursing, respectively. Only postpartum weights were included during the nursing season. The number of body weight measures per female averaged 18.19 ± 10.26 measurements per individual (range: 2–61, n = 4,650 measurements from 468 female replicates). Before statistical modeling, we verified that female body weight and AGD were not correlated during the mating season (r*s* = 0.023; *t* value = 1.948; *p* value = 0.053, n = 160) or during the nursing season (r*s* = 0.002; *t* value = 0.832; *p* value = 0.405, n = 308). This marginally non-significant correlation between female body weight and AGD during the mating season was expected because about 30% of the females have not yet reached their adult body weight (180 g). While females are growing, body weight continues to increase, and morphometric variables, such as AGD, continue to elongate. The correlation between body weight and AGD disappears when females end their growing (i.e., whenever they exceed ~ 180 g)^22^.

### Measures of serum T levels

Blood samples were obtained from all adult and mated females once per season. During the mating season, females were sampled to measure T during the beginning of the sampling period, when most females were in estrus^22^. During the nursing season, females were sampled to measure T during their first capture after parturition, when mated females were in early lactation. Samples were obtained by venipuncture of the saphenous vein, which was punctured with a sterile 14 G needle, allowing ~ 700 μL of blood to drip into a 1.7 mL Eppendorf tube. Each female was punctured only once, and after obtaining the sample hemostasis was performed to stop bleeding. All samples were obtained between 08:00–10:00 am. Blood samples were consistently taken by the same experienced veterinarians. We centrifuged blood samples at 6000 rpm for 10 min. Serum was separated from blood cells and stored at − 20 °C before subsequent analysis. T levels were measured by radioimmunoassay (RIA) for samples from 2009 to 2015, and by enzyme-immunoassay (ELISA) for samples from 2016 to 2019. We used two techniques because in 2015, the RIA equipment was replaced with ELISA equipment due to biosafety reasons. All samples were analyzed in the Endocrinology Laboratory at P. Universidad Católica de Chile. The RIA technique had a detection limit of 0.3 nmol/L, while the ELISA technique had a detection limit of 0.19 nmol/L. All samples were analyzed in duplicate, and the precision of the assay was evaluated by determining the coefficient of intra- and inter-assay variation. For RIA, intra- and inter-assay variation were 7.7% and 9.9%, respectively. For ELISA, intra- and inter-assay variation were 10.6% and 6.39%, respectively. Finally, we used the Welch test to confirm that mean estimates of T measured via RIA (n = 779) and ELISA (n = 677) did not differ significantly (*t* value = 1.09, *df* = 1309.8, *p* value = 0.276).

### Genetic determination of female litter size at weaning

We genotyped a total of 3,619 adults and offspring from 2009 through 2019 (Table S7, supplementary material 5). We estimate that this effort sampled > 90% of breeding adults and offspring each year in the population. Tissue samples (1 × 5 mm ear snips) were taken from each individual and stored in 99% ethanol at 5–6 °C until analysis. DNA was extracted using the Reliaprep DNA animal tissue miniprep system kit (Promega). DNA was eluted in 200 μL of nuclease-free water and stored at − 20 °C. We worked with 10 microsatellite loci, 9 from *O. degus*^78^ and one from *S. cyanus*^79^. These loci were amplified via polymerase chain reaction (PCR), with the following protocol: 15 min at 94 °C for DNA denaturation, 30 cycles of a 1 min denaturation step at 94 °C, followed by 1 min of locus-specific annealing temperature (Table S8, supplementary material 5), 1 min at 72 °C for elongation, and a final elongation step of 10 min at 72 °C. For fragment analysis, the PCR products were mixed in three combinations. Each of these mixes was contrasted with an internal size standard and analyzed using an ABI Prism 3130Xl genetic analyzer and allele sizes were determined using the Genemapper software (Applied Biosystems). All loci amplified successfully and were polymorphic (Table S8, supplementary material 5). Genotypes for all individuals across the years were complete with no missing data. We tested the Hardy–Weinberg (HW) observed and expected heterozygosity for each study year with CERVUS 3.0 software^80^. Deviations from HW expectations were detected in 10 out of 11 years (Table S9, supplementary material 5) and were not the consequence of null allele presence, as all markers were checked for null alleles with MicroChecker software^81^. This finding was expected, because our study population was open, nonpanmitic, and finite^40^. We used the CERVUS 3.0 software^80^ to estimate maternity and paternity. To do so, we examined individual offspring, potential mothers, and potential fathers as trios in the population. Confidence calculations on CERVUS 3.0 were made using the logarithm to the base 10 of the odds score option. All 10 loci selected had a combined exclusion probability of 99.89% for each study year when neither parent was previously known. Based on our estimates of genetic maternity and genetic paternity, we tallied the number of offspring weaned by each female in the population during each year of study. Table S10, supplementary material 5, includes the number of offspring that were or were not assigned to mothers and fathers. For mated females, reproductive success was quantified as the litter size at weaning based on genetic maternity analyses. This variable was measured at weaning when offspring became trappable^35^. In our population, 100% of females reach advanced stages of pregnancy, implying that most if not all females mate and gestate, but not all females wean offspring. Of relevance, we were unable to access the offspring from birth through early weaning (2–3 weeks of age), implying we could not quantify early offspring mortality.

### The number of male mating partners

The number of male mating partners attained by each mated female was determined from tallying the total number of males that fathered one or more offspring weaned by each focal female. We used identity of group members to determine the number of male mating partners that were from the same social group or from other social groups. We computed the total number of male mating partners of each focal female after adding the number of mates within and outside its social group.

### Statistical analysis

We ran three models to test hypothesis 1, which examines the potential effects of AGD, adult serum T levels, and the number of males within the social group on the number of male mating partners attained. To test prediction (i) we ran model 1 (Table 1, model 1), which included n = 160 replicates, representing all mated females sampled during the mating season. To test prediction (ii), we examined model 2 (Table 1, model 2), which included n = 118 replicates, representing all mated females sampled during the mating season, and whose social group included at least one male. To test prediction (iii), we ran model 3 (Table 1, model 3), which included n = 160 replicates, representing all mated females sampled during the mating season. To test hypothesis 2 (i.e., prediction iv), which tested the potential effects of AGD, adult serum T, and the number of male mating partners on litter size at weaning, we ran model 4 (Table 1, model 4), which included n = 160 replicates, representing all mated females sampled during the mating season. To test hypothesis 3 (and prediction v) which tests the potential effects of focal female AGD, adult serum T, and their interaction on litter size at weaning, we ran model 5 (Table 1, model 5). This model included n = 308 replicates, representing all mated females sampled during the nursing season. For completeness, all full models examined to test predictions of hypotheses 1 through 3 included factor interactions among specific predictors included.

We fitted each generalized mixed-effect model to a Poisson error distribution with a log-link function^82^. All models included the year, the social group identity, and female identity as random factors to account for possible variance contribution and repeated measures. Focal female bodyweight was log-transformed due to differences in scaling between variables. Values are reported as means ± SD. Model fits were assessed through examining quantile residual dispersion. Best fitting models were selected through model selection routines based on their difference in AICc and weight^83^. All statistical analyses were performed in R 4.3.1^84^. Generalized mixed models (GLMM) were fitted with the package LME4 1.1–34^85^, and DHARMa 0.4.6^86^ and MuMIn 1.47.5^87^ packages were used to perform model fit and model selection routines, respectively.

### Ethical note

Authors confirm that animal handling techniques and all the protocols used in this study followed the Guide of the American Society of Mammalogists for the use of wild animals in research^88^ and are in accordance with ARRIVE guidelines^89^. Authors confirm that all protocols implemented during this study were approved by the Scientific Ethical Committee for the Care of Animals and the Environment, of the Pontificia Universidad Católica de Chile (CBB-155, 2012 resolution, supervised and approved 03/03/2015, CBB-170509009 resolution, supervised and approved 08/2020), and by the Bioethics Committee for Use of Animals in Research of the Universidad Austral de Chile (DID-03/09 resolution, supervised and approved 10/06/2009), and followed the Chilean Ethical Legislation (Permits 1–31/2009, 3881/2012, 2826/2013, 6975/2017 and 2890/2019, by the Servicio Agricola y Ganadero). Blood sampling was performed by two well-trained veterinarians.

## Electronic supplementary material

Below is the link to the electronic supplementary material.

Supplementary Material 1

Supplementary Material 2

Supplementary Material 3

Supplementary Material 4

Supplementary Material 5

## Data Availability

Analyses reported in this article can be reproduced using the data provided in: https://datadryad.org/stash/dataset/doi:10.5061/dryad.5mkkwh7g6
