# Supplementary material for: Organizational effects of testosterone on the number of mating partners and reproductive success in females of a social rodent
Source: Sci Rep. 2025 Jul 1;15:22411. doi: 10.1038/s41598-025-03708-y (PMC12215531; doi:10.1038/s41598-025-03708-y)
Supplement: Supplementary file 1 — Supplementary Material 1 [file 41598_2025_3708_MOESM1_ESM.docx]

**Supplementary Material 1 – Model selection**

**Table S1 Selected sub-model after selection routine conducted from Model 1 (total number of male mating partners attained).** Year of study, the social group ID (SGID), and female identity (female ID) were included as random factors. The selected generalized mixed-effects model is reported first in bold typing.

| **Sub-model** | **Model** | **df** | **AIC** | **Delta** | **Weight** |
| --- | --- | --- | --- | --- | --- |
| **1.1** | **Log focal female body weight** | **5** | **500.56** | **0.00** | **0.43** |
| 1.2 | Focal female AGD + log focal female body weight | 6 | 502.62 | 2.06 | 0.15 |
| 1.3 | Log focal female body weight + n° of males within social group | 6 | 502.70 | 2.14 | 0.15 |
| 1.4 | Log focal female body weight + focal female serum T | 6 | 502.72 | 2.16 | 0.15 |
| 1.5 | NULL | 4 | 503.17 | 2.61 | 0.12 |

**Table S2 Selected sub-model after selection routine conducted from Model 2 (number of male mating partners attained within social group).** Year of study, the social group ID (SGID), and female identity (female ID) were included as random factors. The selected generalized mixed-effects model is reported first in bold typing.

| **Sub-model** | **Model** | **df** | **AIC** | **Delta** | **Weight** |
| --- | --- | --- | --- | --- | --- |
| **2.1** | **Log focal female body weight + n° of males within social group** | **6** | **228.04** | **0.00** | **0.32** |
| 2.2 | N° of males within social group | 5 | 228.31 | 0.26 | 0.28 |
| 2.3 | Focal female AGD + log focal female body weight + n° of males within social group | 7 | 229.65 | 1.61 | 0.14 |
| 2.4 | Focal female AGD + n° of males within social group | 6 | 229.93 | 1.89 | 0.13 |
| 2.5 | Log focal female body weight + n° of males within social group + focal female serum T | 7 | 229.97 | 1.92 | 0.12 |

**Table S3 Selected sub-model after selection routine conducted from Model 3 (number of male mating partners attained outside social group).** Year of study, the social group ID (SGID), and female identity (female ID) were included as random factors. The selected generalized mixed-effects model is reported first in bold typing.

| **Sub-model** | **Model** | **df** | **AIC** | **Delta** | **Weight** |
| --- | --- | --- | --- | --- | --- |
| **3.1** | **Log focal female body weight + n° of males within social group** | **6** | **488.74** | **0.00** | **0.32** |
| 3.2 | N° of males within social group | 5 | 489.05 | 0.31 | 0.27 |
| 3.3 | Focal female AGD + n° of males within social group | 6 | 490.11 | 1.37 | 0.16 |
| 3.4 | Focal female AGD + log focal female body weight + n° of males within social group | 7 | 490.30 | 1.56 | 0.14 |
| 3.5 | Focal female AGD + n° of males within social group + (focal female AGD * n° of males within social group) | 8 | 490.84 | 2.10 | 0.11 |

**Table S4 Selected sub-model after selection routine conducted from Model 4** (**litter size at weaning).** Year of study, the social group ID (SGID), and female identity (female ID) were included as random factors. The selected generalized mixed-effects model is reported first in bold typing.

| **Sub-model** | **Model** | **df** | **AIC** | **Delta** | **Weight** |
| --- | --- | --- | --- | --- | --- |
| **4.1** | **Log focal female body weight + n° of male mating partners attained** | **6** | **664.90** | **0.00** | **0.39** |
| 4.2 | Focal female AGD + log focal female body weight + n° of male mating partners attained + (focal female AGD * n° of male mating partners attained) | 8 | 666.08 | 1.18 | 0.21 |
| 4.3 | Focal female AGD + log focal female body weight + n° of male mating partners attained | 7 | 666.34 | 1.44 | 0.19 |
| 4.4 | Log focal female body weight + n° of male mating partners attained + focal female serum T | 7 | 666.96 | 2.06 | 0.14 |
| 4.5 | Focal female AGD + log focal female body weight + n° of male mating partners attained + focal female serum T + (focal female AGD * n° of male mating partners attained) | 9 | 668.29 | 3.39 | 0.07 |

**Table S5 Selected sub-model after selection routine conducted from Model 5 (litter size at weaning).** Year of study, the social group ID (SGID), and female identity (female ID) were included as random factors. The selected generalized mixed-effects model is reported first in bold typing.

| **Sub-model** | **Model** | **df** | **AIC** | **Delta** | **Weight** |
| --- | --- | --- | --- | --- | --- |
| **5.1** | **Focal female AGD + log focal female body weight** | **6** | **1394.04** | **0.00** | **0.48** |
| 5.2 | Focal female AGD + log focal female body weight + focal female serum T | 7 | 1395.87 | 1.82 | 0.19 |
| 5.3 | Log focal female body weight | 5 | 1396.08 | 2.04 | 0.17 |
| 5.4 | Focal female AGD + log focal female body weight + focal female serum T + (focal female AGD * focal female serum T) | 8 | 1397.78 | 3.74 | 0.07 |
| 5.5 | Log focal female body weight + focal female serum T | 6 | 1397.84 | 3.80 | 0.07 |
