# Supplementary material for: Organizational effects of testosterone on the number of mating partners and reproductive success in females of a social rodent
Source: Sci Rep. 2025 Jul 1;15:22411. doi: 10.1038/s41598-025-03708-y (PMC12215531; doi:10.1038/s41598-025-03708-y)
Supplement: Supplementary file 2 — Supplementary Material 2 [file 41598_2025_3708_MOESM2_ESM.docx]

**Supplementary Material 2 – Social Groups**

**Table S6.** Number of burrow systems, number of days that each burrow system was trapped, number of radiocollared degus, and mean (± SD) number of nighttime telemetry locations per radiocollared degu during each season (winter, spring) and year of study (2009-2019) at Estacion Experimental German Greve Silva. *Number in parentheses indicates the number of radiocollared degus that were radiotracked to their burrows during at least 8 nights and that were confirmed to be alive during telemetry.

| **Year** | **Mating season (winter)** | | | |  | **Nursing season (spring)** | | | |
| --- | --- | --- | --- | --- | --- | --- | --- | --- | --- |
|  | #burrow systems monitored | #days trapped | #radiocollared degus* | Nighttime locations (mean ±SE) per degu |  | #burrow systems monitored | #days trapped | #radiocollared degus* | Nighttime locations (mean ±SE) per degu |
| 2009 | 50 | 35 | 22 (17) | 16.7 ± 0.4 |  | 44 | 44 | 32 (31) | 15.8 ± 0.1 |
| 2010 | 42 | 30 | 20 (17) | 12.6 ± 0.2 |  | 40 | 42 | 17 (17) | 14.9 ± 0.1 |
| 2011 | 54 | 37 | 21 (21) | 20.9 ± 1.5 |  | 68 | 48 | 34 (33) | 19.6 ± 0.9 |
| 2012 | 54 | 35 | 13 (10) | 13.5 ± 0.7 |  | 58 | 50 | 33 (22) | 14.8 ± 0.5 |
| 2013 | 62 | 36 | 39 (36) | 16.9 ± 0.3 |  | 58 | 44 | 37 (37) | 18.6 ± 0.4 |
| 2014 | 46 | 38 | 28 (25) | 14.5 ± 0.2 |  | 50 | 54 | 33 (29) | 22.1 ± 0.9 |
| 2015 | 53 | 36 | 35 (31) | 21.0 ± 0.5 |  | 59 | 63 | 40 (38) | 20.5 ± 0.6 |
| 2016 | 48 | 35 | 37 (36) | 15.8 ± 0.4 |  | 50 | 59 | 47 (44) | 19.3 ± 0.2 |
| 2017 | 57 | 35 | 47 (43) | 18.3 ± 0.3 |  | 54 | 55 | 50 (47) | 21.4 ± 0.6 |
| 2018 | 56 | 39 | 58 (50) | 25.1 ± 0.6 |  | 53 | 53 | 45 (44) | 25.1 ± 0.5 |
| 2019 | 46 | 38 | 34 (28) | 19.2 ± 0.8 |  | 42 | 56 | 27 (25) | 24.5 ± 0.6 |
