# Supplementary material for: Organizational effects of testosterone on the number of mating partners and reproductive success in females of a social rodent
Source: Sci Rep. 2025 Jul 1;15:22411. doi: 10.1038/s41598-025-03708-y (PMC12215531; doi:10.1038/s41598-025-03708-y)
Supplement: Supplementary file 3 — Supplementary Material 3 [file 41598_2025_3708_MOESM3_ESM.docx]

**Supplementary Material 3 – Details of how social group membership was determined.**

Determining social group membership from trapping and telemetry data involved multiple steps. First, we created two matrices in Microsoft Excel, one for trapping data and one for telemetry data. Second, we calculate pairwise “simple ratio” association indices separately (independently) for trapping and telemetry data using SOCPROG 2.5 software^1^. This association was quantified dividing the number of days or evenings that two individuals were located in the same burrow system by the total number of days or evenings they were either captured or tracked by telemetry. This allowed us to obtain trapping and/or telemetry association for each pair of individuals, accounting for both spatial and temporal overlap.

Third, based on these associations, we produced dendrograms for visual inspection of spatiotemporal overlap using SOCPROG 2.5 (Fig. S1). We selected the maximum modularity criteria in SOCPROG to cut off the dendrogram. We confirmed the correlation between association and the level of clustering in the dendrogram output with the cophenetic correlation coefficient^1^.

Fourth, we used these dendrograms to assign degus to ‘preliminary’ social groups. Initially, only social groups with an average association greater than 0.1 (i.e. 10% overlap of trapping/telemetry locations) in the SOCPROG cluster analysis were considered in assigning preliminary social groups^2^. We prioritized dendrograms based on telemetry because: (i) telemetry provides precise locations of individuals because individuals remain in burrows at night, and (ii) whereas trapping success among individuals during early daytime activity varies, telemetry data were collected from all radio-collared individuals on the same evenings. Final social units were determined after closer inspection of associations among individuals and spatial overlap at burrow systems based on telemetry and trapping during early daytime activity. Weak associations among individuals in a dendrogram occurred when they were rarely captured together on the same night, or when an individual shared a burrow system on one or two nights that was not regularly used by other group members. We removed individuals with these weak associations.


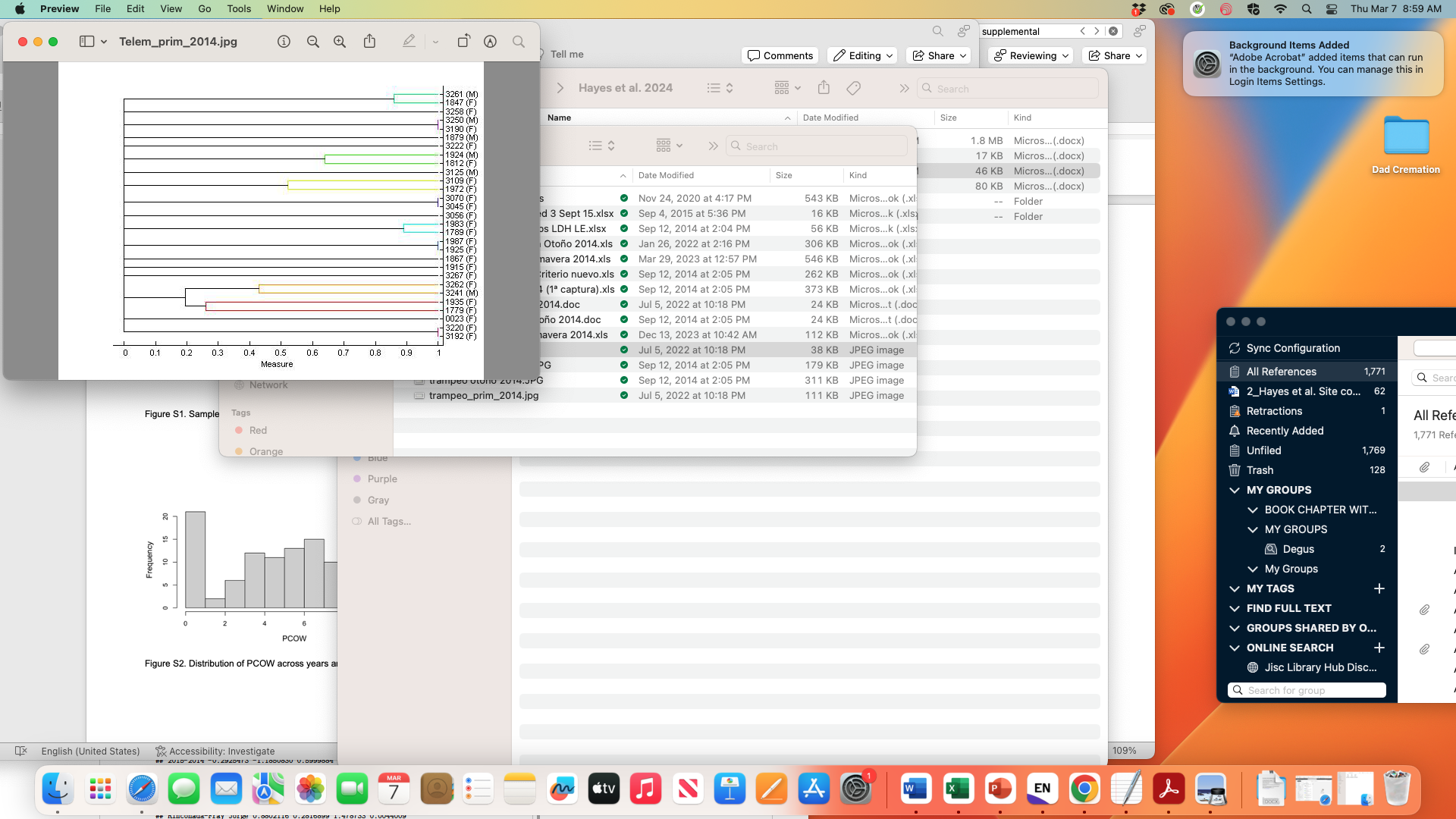


**Figure S1.** Sample dendrogram produced by SOCPROG 2.5 software, with data from spring 2014. Each cluster of a color indicates a social group. In this figure, ten social groups are recognized which, in this case, correspond to female pairs or female-male pairs. Four-digit numbers are animal identification numbers. F=female. M=male.

**References**

1. Whitehead, H. SOCPROG programs: analyzing animal social structures. *Behav. Ecol. Sociobiol*. **63**, 765‒778 (2009).

2. Hayes, L.D., Chesh, A.S., Castro, R.A., Tolhuysen, L.O., Burger, J.R., Bhattacharjee, J., & Ebensperger, L.A. Fitness consequences of group living in the degu *Octodon degus*, a plural breeder rodent with communal care. *Anim. Behav.* **78**, 131‒139 (2009).
