# Supplementary material for: Organizational effects of testosterone on the number of mating partners and reproductive success in females of a social rodent
Source: Sci Rep. 2025 Jul 1;15:22411. doi: 10.1038/s41598-025-03708-y (PMC12215531; doi:10.1038/s41598-025-03708-y)
Supplement: Supplementary file 4 — Supplementary Material 4 [file 41598_2025_3708_MOESM4_ESM.docx]

**
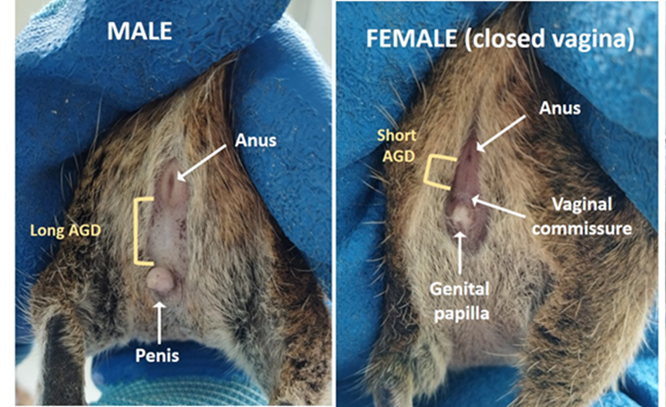
****Supplementary Material 4 – Female anogenital distance (AGD) measurement and distribution**

**Figure S2.** Picture of adult male and female anogenital distance (AGD) in degus. In the left side male AGD, in the right side female AGD. In the male picture, the anus and penis are distinguished, and the AGD is significantly longer, compared to the AGD of females. In the female picture, the anus, the genital papilla, and the vaginal commissure are distinguished, and the AGD is significantly shorter, compared to the AGD of males. The brackets in soft yellow indicate the reference points for measuring AGD. In males, the AGD measurement is from the ventral commissure of the anus to the base of the penis. In females, the AGD measurement is from the ventral commissure of the anus to the vaginal commissure. In females we measured AGD length only when females had a closed (non-perforated) vagina.

**
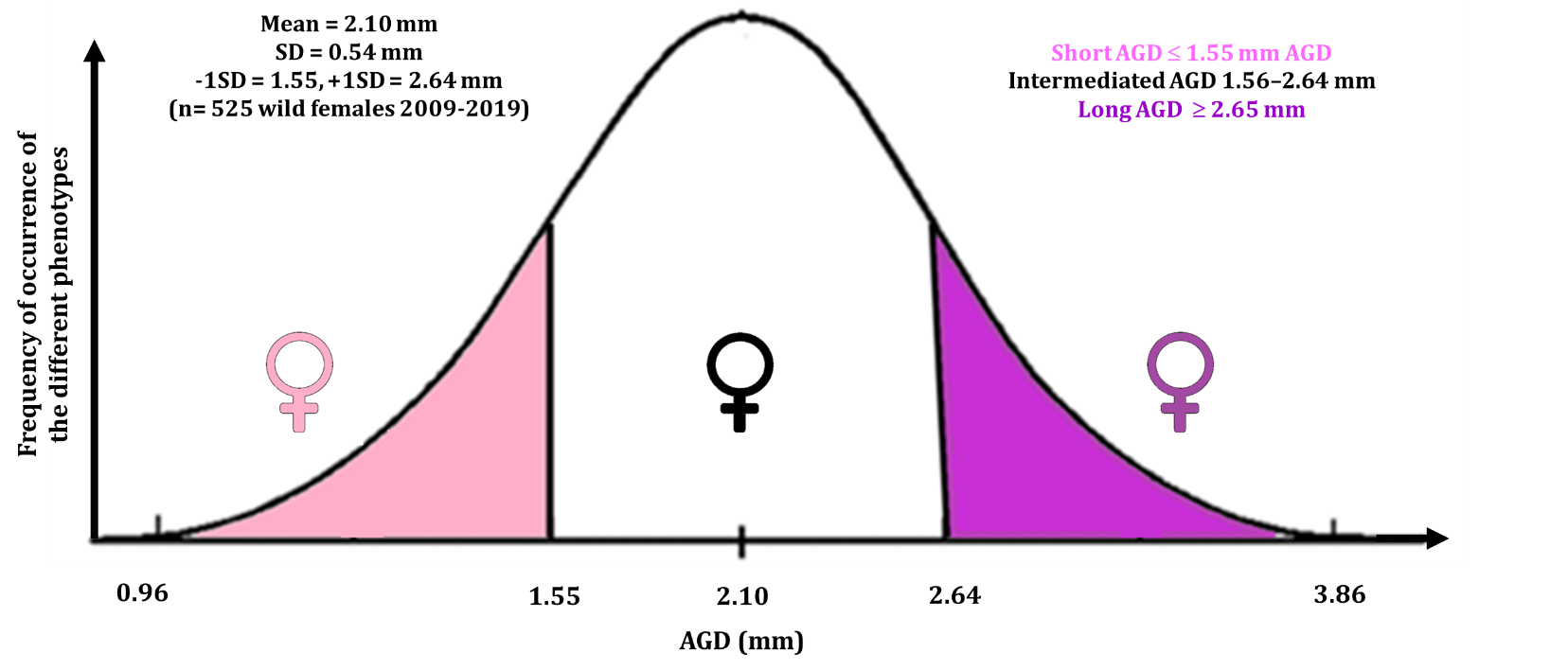
**

**Figure S3.** Female degus AGD distribution in mm, during winter and spring season. During both seasons, the AGD distribution of females corresponds a normal distribution. The limits to define females with short AGD and long AGD, are obtained by subtracting and adding a standard deviation to the population mean. Short and long AGD females represent the 15-20% of population, being the infrequent phenotypes. Intermediated AGD females represent the 60-65% of population being the frequent phenotype. This classification is only to describe different AGD phenotypes when discussing our results, as AGD was used as a continuous predictor for all statistical analyses.
