# Supplementary material for: Organizational effects of testosterone on the number of mating partners and reproductive success in females of a social rodent
Source: Sci Rep. 2025 Jul 1;15:22411. doi: 10.1038/s41598-025-03708-y (PMC12215531; doi:10.1038/s41598-025-03708-y)
Supplement: Supplementary file 5 — Supplementary Material 5 [file 41598_2025_3708_MOESM5_ESM.doc]

**Supplementary Material 5 – Genetic Methods**

**Table S7.** Total number (n = 3619) of genotyped adult and offspring degus (2009-2019).

| **Year of study** | **Number of adult females** | **Number of adult males** | **Number of female offspring** | **Number of male offspring** | **Total** |
| --- | --- | --- | --- | --- | --- |
| 2009 | 47 | 49 | 85 | 117 | 298 |
| 2010 | 10 | 26 | 18 | 30 | 84 |
| 2011 | 47 | 34 | 83 | 88 | 252 |
| 2012 | 30 | 27 | 62 | 64 | 183 |
| 2013 | 52 | 48 | 135 | 149 | 384 |
| 2014 | 71 | 71 | 99 | 86 | 327 |
| 2015 | 78 | 87 | 149 | 140 | 454 |
| 2016 | 130 | 100 | 246 | 228 | 704 |
| 2017 | 73 | 45 | 173 | 174 | 465 |
| 2018 | 77 | 62 | 102 | 82 | 323 |
| 2019 | 45 | 38 | 31 | 31 | 145 |
| Total | 660 | 587 | 1183 | 1189 | 3619 |

**Table S8.** Sequence, annealing temperature (Ta), size, and number of alleles of 10 microsatellite loci used to genotype degu adults and offspring.

| **Locus** | **Repeat motif** | **Primer sequence (5'-3')** | **Ta (C°)** | **PCR product size (bp)** | **Number of alleles** | **GenBank Accession n°** |
| --- | --- | --- | --- | --- | --- | --- |
| OCDE1 | (CTTT)_7CTCT_(CTTT)_10_ | F: VIC-CTAGGTGCCAGAGACCCTTG | 60 | 152-184 | 9 | FJ418930 |
|  |  | R: CAAAGACCCTGGGTTCAATC |  |  |  |  |
| OCDE2 | (CA)_13_ | F: VIC-GTTCGAGCTGCCTAGTGAGG | 64 | 200-214 | 7 | FJ418931 |
|  |  | R: ACTGGACATGGTGGTGTGTG |  |  |  |  |
| OCDE5 | (GAAA)_11GAGA_(GAAA)_7_ | F: FAM-CAAAGACCCTGGGTTCAATC | 58 | 196-228 | 9 | FJ418934 |
|  |  | R: CATGATTGAGCTTGCCTCTG |  |  |  |  |
| OCDE7 | (GAAA)_13_(GA)_4_* | F: FAM-CAAGCTTGTCAAAGCACAGG | 64 | 191-229 | 17 | FJ418936 |
|  |  | R: GGCAGAAAATTCTGGACAGG |  |  |  |  |
| OCDE9 | (GA)_23_ | F: FAM-CATGTAGTTTTCCAGGCACT | 58 | 169-197 | 13 | FJ418938 |
|  |  | R: TTCCTCCACTTTCTGACAAT |  |  |  |  |
| OCDE10 | (TG)_13_ | F: NED-AAGGCAGCAGTTGGGAGAACAA | 64 | 157-185 | 10 | FJ418939 |
|  |  | R: TGAGATTGTCCTTTGAGTCCACATGA |  |  |  |  |
| OCDE11 | (CA)_5TATA_(CA)_4_ | F: PET-TAGGAAGGAAAGGAGCTGGA | 58 | 164-180 | 8 | FJ418940 |
|  | GAGACAAATA(CA)_20_ | R: CAACAAGCTCGGGTGATTTA |  |  |  |  |
| OCDE12 | (GT)_15_ | F: PET-GCAGAGCTAAGGACTAAAGGTTCCA | 62 | 174-224 | 19 | FJ418941 |
|  |  | R: CCAAGTTGCTAAGAGGTCCCTTG |  |  |  |  |
| OCDE14 | (GT)_20_(TG)_2_ | F: FAM-GCTCTGGGGGCAATCAATATTCT | 58 | 150-174 | 12 | FJ418943 |
|  |  | R: AAACCACTACTTCTGCACTGTTCCA |  |  |  |  |
| SCY3 | (CA)_20_ | F: NED-AAGTTGAGGCTAGTTGTTTG | 52 | 125-151 | 12 | AF250221 |
|  |  | R: GATCACAGGCACCACATAC |  |  |  |  |

*OCDE7 was originally described as (GAAA)_15_, a four motif repeat (Quan et al., 2009). However, the forward and reversal sequencing of this marker in our Molecular Facility revealed this is two motif repeat marker.

**Table S9.** Analysis of Hardy-Weinberg (HW) expectations for each locus within each study year (2009 to 2019). Data include the number of alleles (NA), observed heterozygosity (H_obs_), expected heterozygosity (H_exp_), Hardy-Weinberg p-value after Bonferroni corrections, and significant deviations from HW expectations. Key to statistical significance: NS = not significant * = significant at p<0.05** = significant at p<0.01, and *** = significant at p<0.001.

| **Locus** | **2009** | | | | |  | **2010** | | | | |  | **2011** | | | | |
| --- | --- | --- | --- | --- | --- | --- | --- | --- | --- | --- | --- | --- | --- | --- | --- | --- | --- |
|  | **N_A_** | **H_obs_** | **H_exp_** | **p-value** | **HW** |  | **NA** | **Hobs** | **Hexp** | **p-value** | **HW** |  | **NA** | **Hobs** | **Hexp** | **p-value** | **HW** |
| OCDE1 | 9 | 0.893 | 0.843 | 0.166 | NS |  | 8 | 0.798 | 0.783 | 0.240 | NS |  | 9 | 0.869 | 0.839 | 0.694 | NS |
| OCDE2 | 7 | 0.829 | 0.803 | < 0.001 | *** |  | 7 | 0.798 | 0.794 | 0.632 | NS |  | 7 | 0.726 | 0.789 | 0.160 | NS |
| OCDE5 | 9 | 0.872 | 0.843 | 0.151 | NS |  | 8 | 0.702 | 0.789 | 0.118 | NS |  | 9 | 0.845 | 0.834 | 0.854 | NS |
| OCDE7 | 13 | 0.842 | 0.874 | < 0.001 | *** |  | 13 | 0.833 | 0.840 | 0.406 | NS |  | 13 | 0.853 | 0.881 | 0.094 | NS |
| OCDE9 | 13 | 0.856 | 0.866 | 0.027 | NS |  | 11 | 0.917 | 0.860 | < 0.001 | *** |  | 12 | 0.869 | 0.866 | 0.068 | NS |
| OCDE10 | 10 | 0.795 | 0.788 | 0.312 | NS |  | 8 | 0.690 | 0.721 | 0.652 | NS |  | 9 | 0.766 | 0.735 | 0.336 | NS |
| OCDE11 | 6 | 0.779 | 0.761 | 0.012 | NS |  | 7 | 0.857 | 0.791 | 0.021 | NS |  | 7 | 0.786 | 0.823 | 0.019 | NS |
| OCDE12 | 14 | 0.822 | 0.853 | 0.007 | NS |  | 15 | 0.905 | 0.899 | 0.899 | NS |  | 16 | 0.861 | 0.862 | 0.010 | NS |
| OCDE14 | 9 | 0.802 | 0.795 | 0.049 | NS |  | 9 | 0.774 | 0.805 | < 0.001 | *** |  | 9 | 0.750 | 0.753 | 0.391 | NS |
| SCY3 | 9 | 0.822 | 0.800 | 0.895 | NS |  | 10 | 0.786 | 0.825 | 0.030 | NS |  | 11 | 0.841 | 0.826 | 0.087 | NS |

**Table S9.** Continuation.

| **Locus** | **2012** | | | | |  | **2013** | | | | |  | **2014** | | | | |
| --- | --- | --- | --- | --- | --- | --- | --- | --- | --- | --- | --- | --- | --- | --- | --- | --- | --- |
|  | **NA** | **Hobs** | **Hexp** | **p-value** | **HW** |  | **NA** | **Hobs** | **Hexp** | **p-value** | **HW** |  | **NA** | **Hobs** | **Hexp** | **p-value** | **HW** |
| OCDE1 | 9 | 0.798 | 0.854 | < 0.001 | *** |  | 9 | 0.859 | 0.848 | 0.006 | NS |  | 9 | 0.838 | 0.854 | 0.073 | NS |
| OCDE2 | 7 | 0.776 | 0.799 | 0.103 | NS |  | 7 | 0.745 | 0.789 | 0.001 | * |  | 6 | 0.758 | 0.794 | 0.262 | NS |
| OCDE5 | 9 | 0.831 | 0.855 | 0.003 | * |  | 9 | 0.862 | 0.846 | < 0.001 | ** |  | 9 | 0.847 | 0.853 | 0.034 | NS |
| OCDE7 | 14 | 0.814 | 0.855 | 0.048 | NS |  | 14 | 0.797 | 0.857 | < 0.001 | *** |  | 15 | 0.78 | 0.836 | 0.011 | NS |
| OCDE9 | 11 | 0.869 | 0.855 | 0.011 | NS |  | 13 | 0.862 | 0.852 | 0.127 | NS |  | 13 | 0.859 | 0.85 | 0.039 | NS |
| OCDE10 | 9 | 0.798 | 0.775 | 0.676 | NS |  | 9 | 0.742 | 0.750 | 0.004 | * |  | 10 | 0.783 | 0.774 | 0.107 | NS |
| OCDE11 | 7 | 0.787 | 0.808 | 0.016 | NS |  | 7 | 0.794 | 0.790 | < 0.001 | ** |  | 8 | 0.774 | 0.792 | < 0.001 | ** |
| OCDE12 | 15 | 0.880 | 0.877 | 0.029 | NS |  | 16 | 0.906 | 0.893 | 0.001 | * |  | 14 | 0.902 | 0.894 | 0.725 | NS |
| OCDE14 | 10 | 0.716 | 0.736 | 0.079 | NS |  | 12 | 0.766 | 0.767 | 0.202 | NS |  | 11 | 0.777 | 0.797 | < 0.001 | ** |
| SCY3 | 11 | 0.923 | 0.881 | < 0.001 | ** |  | 11 | 0.893 | 0.871 | 0.001 | * |  | 11 | 0.865 | 0.878 | < 0.001 | *** |

**Table S9.** Continuation.

| **Locus** | **2015** | | | | |  | **2016** | | | | |  | **2017** | | | | |
| --- | --- | --- | --- | --- | --- | --- | --- | --- | --- | --- | --- | --- | --- | --- | --- | --- | --- |
|  | **NA** | **Hobs** | **Hexp** | **p-value** | **HW** |  | **NA** | **Hobs** | **Hexp** | **p-value** | **HW** |  | **NA** | **Hobs** | **Hexp** | **p-value** | **HW** |
| OCDE1 | 9 | 0.833 | 0.842 | 0.004 | * |  | 9 | 0.838 | 0.837 | 0.186 | NS |  | 9 | 0.847 | 0.858 | 0.583 | NS |
| OCDE2 | 6 | 0.744 | 0.767 | 0.211 | NS |  | 6 | 0.743 | 0.799 | < 0.001 | *** |  | 6 | 0.746 | 0.77 | 0.016 | NS |
| OCDE5 | 9 | 0.841 | 0.842 | 0.004 | * |  | 9 | 0.830 | 0.836 | 0.103 | NS |  | 9 | 0.843 | 0.858 | 0.668 | NS |
| OCDE7 | 15 | 0.835 | 0.851 | 0.04 | NS |  | 15 | 0.832 | 0.850 | < 0.001 | ** |  | 14 | 0.796 | 0.818 | 0.002 | * |
| OCDE9 | 12 | 0.866 | 0.845 | 0.583 | NS |  | 12 | 0.839 | 0.846 | 0.001 | ** |  | 12 | 0.886 | 0.861 | 0.002 | * |
| OCDE10 | 10 | 0.802 | 0.808 | 0.005 | NS |  | 10 | 0.803 | 0.799 | 0.047 | NS |  | 10 | 0.798 | 0.786 | 0.114 | NS |
| OCDE11 | 8 | 0.813 | 0.792 | 0.782 | NS |  | 8 | 0.778 | 0.796 | 0.068 | NS |  | 8 | 0.794 | 0.804 | 0.033 | NS |
| OCDE12 | 17 | 0.921 | 0.903 | 0.027 | NS |  | 19 | 0.866 | 0.892 | < 0.001 | *** |  | 20 | 0.886 | 0.883 | < 0.001 | *** |
| OCDE14 | 9 | 0.791 | 0.788 | 0.006 | NS |  | 10 | 0.778 | 0.782 | 0.001 | * |  | 13 | 0.811 | 0.851 | < 0.001 | *** |
| SCY3 | 12 | 0.883 | 0.881 | 0.051 | NS |  | 12 | 0.864 | 0.865 | 0.017 | NS |  | 11 | 0.877 | 0.876 | < 0.001 | ** |

**Table S9.** Continuation.

| **Locus** | **2018** | | | | |  | **2019** | | | | |
| --- | --- | --- | --- | --- | --- | --- | --- | --- | --- | --- | --- |
|  | **N_A_** | **H_obs_** | **H_exp_** | **p-value** | **HW** |  | **N_A_** | **H_obs_** | **H_exp_** | **p-value** | **HW** |
| OCDE1 | 9 | 0.853 | 0.853 | 0.341 | NS |  | 9 | 0.766 | 0.855 | 0.009 | NS |
| OCDE2 | 7 | 0.752 | 0.766 | 0.107 | NS |  | 7 | 0.828 | 0.780 | 0.038 | NS |
| OCDE5 | 9 | 0.842 | 0.852 | 0.476 | NS |  | 9 | 0.772 | 0.854 | 0.004 | * |
| OCDE7 | 13 | 0.805 | 0.833 | 0.004 | * |  | 13 | 0.890 | 0.849 | 0.029 | NS |
| OCDE9 | 11 | 0.901 | 0.842 | < 0.001 | ** |  | 12 | 0.890 | 0.843 | 0.262 | NS |
| OCDE10 | 10 | 0.759 | 0.768 | 0.786 | NS |  | 9 | 0.676 | 0.675 | 0.185 | NS |
| OCDE11 | 8 | 0.780 | 0.780 | 0.009 | NS |  | 8 | 0.793 | 0.756 | 0.271 | NS |
| OCDE12 | 17 | 0.864 | 0.877 | 0.013 | NS |  | 14 | 0.876 | 0.874 | 0.189 | NS |
| OCDE14 | 13 | 0.786 | 0.824 | 0.053 | NS |  | 13 | 0.80 | 0.811 | 0.889 | NS |
| SCY3 | 11 | 0.861 | 0.875 | < 0.001 | *** |  | 11 | 0.869 | 0.878 | 0.260 | NS |

**Table S10.** Number of genotyped offspring assigned to a candidate mother and father, assigned to a candidate mother only, assigned to a candidate father only, and unassigned (2009-2019).

| **Year of study** | **Number of offspring assigned to a candidate mother & father** | **Number of offspring assigned to a candidate mother only** | **Number of offspring assigned to a candidate father only** | **Number of offspring unassigned** | **Total** |
| --- | --- | --- | --- | --- | --- |
| 2009 | 178 | 3 | 0 | 22 | 203 |
| 2010 | 25 | 0 | 0 | 23 | 48 |
| 2011 | 98 | 7 | 0 | 66 | 171 |
| 2012 | 77 | 1 | 0 | 48 | 126 |
| 2013 | 242 | 10 | 0 | 32 | 284 |
| 2014 | 162 | 9 | 0 | 14 | 185 |
| 2015 | 246 | 15 | 0 | 28 | 289 |
| 2016 | 432 | 11 | 0 | 32 | 475 |
| 2017 | 256 | 15 | 0 | 76 | 347 |
| 2018 | 167 | 6 | 0 | 11 | 184 |
| 2019 | 60 | 1 | 0 | 1 | 62 |
| **Total** | 1931 | 79 | 0 | 363 | 2374 |
